# The regulatory function of social referencing in preschoolers with Down syndrome or Williams syndrome

**DOI:** 10.1186/1866-1955-5-2

**Published:** 2013-02-13

**Authors:** Angela John Thurman, Carolyn B Mervis

**Affiliations:** 1 Department of Psychological and Brain Sciences, University of Louisville, Louisville, KY, 40292, USA; 2 MIND Institute, Department of Psychiatry and Behavioral Sciences, University of California, UC Davis, 2825 50th Street, Room 2101, Sacramento, CA, 95817, USA

**Keywords:** Down syndrome, Eye contact, Emotional responsivity, Gaze following, Intellectual disability, Joint attention, Social referencing, Williams-Beuren syndrome

## Abstract

**Background:**

An important developmental task is to learn to recognize another person as a source of information and to utilize this information as a method of learning about the surrounding world. This socially guided form of learning, referred to as social referencing, is critical for the development of children’s understanding of other people, themselves and their surrounding world. In the present project, the regulatory function of social referencing was examined in two genetic disorders that are characterized by differing patterns of socio-cognitive development: Down syndrome (DS) and Williams syndrome (WS).

**Methods:**

Participants were 20 children with DS and 20 children with WS aged 42 to 71 months, matched on chronological age and gender. Each child participated in four studies: one study in which we examined performance in a social referencing paradigm and three studies in which we considered performance on tasks designed to tap each of three component abilities (initiating eye contact, gaze following and emotional responsivity) important for success in social referencing.

**Results:**

The majority of children in both groups demonstrated positive behavioral responses regarding the stimulus in the Social Referencing task when the adult communicated a joyful message but did not regulate their own behavior in accordance with the adult’s expression of fear. Between-group differences were observed in both conditions, with most differences indicating more advanced socio-communicative competence for children with DS than for children with WS even though the overall intellectual abilities and receptive language abilities of the children with WS were significantly higher than were those of the children with DS. The results of follow-up studies indicated that children with DS were more likely to initiate eye contact (unsolicited) and to follow another person’s gaze in triadic situations than were children with WS. Neither group regulated their behavior in response to expressions of fear.

**Conclusions:**

These findings provide new insight into the development of the social cognitive phenotypes associated with DS and WS. These social cognitive differences found during the preschool years likely contribute to the differing phenotypes observed later in life between individuals with DS and individuals with WS.

## Background

The social referencing process, a socially guided method of learning, is an important way in which a child learns about his or her environment and how to behave [1]. Consider the following situation: A child encounters a novel object, turns to his/her mother, and sees that she is attending to the same object and smiling. In turn, the child moves toward the object and begins to explore it; that is, the child used his/her mother’s reaction to guide his/her own behavioral response. Similarly, parents often use emotional expressions to communicate approval or disapproval of the child’s behavior as a method of teaching the child socially-appropriate behavioral responses. As shown in these examples, the social referencing process involves an interactive social situation in which one person uses another’s interpretation to form his or her own understanding of that situation [1-3]. The ability to recognize a person as an intentional agent and to interpret a person's actions/behaviors in terms of his or her underlying mental states involves a complex interweaving of cognitive, affective and personality factors. These abilities are important for psychological growth and effective functioning within the environment as they facilitate children's learning about people and the surrounding world [4].

A considerable amount of research has been conducted examining social referencing by typically developing (TD) children, using a variety of methodological approaches. These results indicate that, at around 12 months of age, TD children will modify their proximity to the “referent” and the “referee,” affective responses and looking behavior, in accordance with another person’s appraisal of an ambiguous situation [3,5]. It is important to note that this process requires the child to coordinate his or her attention between an object and the adult (initiate eye contact), to map the adult’s reaction to its source (follow gaze), and to comprehend the communicative significance of the adult’s emotionally valenced message. To date, although these abilities have been studied individually, no single study or series of studies has considered the specific roles of these three abilities in relation to the regulatory function of social referencing.

Significant contributions toward understanding of the social referencing process itself, as well as toward understanding of socio-cognitive development, may be made by examining the social referencing process in children with developmental delays of known genetic etiology. A syndrome-specific approach to the examination of the social referencing process offers the opportunity to consider the influence of specific abilities fundamental to the development of social cognition, providing information on genetic or other mechanisms associated with socio-cognitive abilities. The purpose of the present project was to examine the regulatory function of social referencing in two genetic syndromes characterized by differing socio-cognitive phenotypes [Down syndrome (DS) and Williams syndrome (WS)] and to evaluate each of the three abilities fundamental to the social referencing process (initiating eye contact, gaze following and emotional responsivity) individually to clarify which specific skills are likely facilitating and/or impeding the social referencing process for children with each syndrome.

### The behavioral phenotypes: Down syndrome and Williams syndrome

DS, with a prevalence of 1 in 733 live births [6], is caused by an extra copy of the long arm of chromosome 21 (trisomy 21) and is the most common genetic cause of intellectual disability (ID). DS is associated with dysmorphic facial characteristics, congenital heart defects, short stature, hypotonia and immune and endocrine system abnormalities [7]. Despite significant expressive language difficulties, individuals with DS demonstrate a relative strength in socio-communicative abilities that may be used to compensate for limitations in other areas [8,9]. Individuals with DS are frequently described as having charming personalities [10], being affectionate and loveable, and getting along well with others [11]. Difficulties in social functioning have been noted as well. Children with DS are often characterized as demonstrating sudden changes in mood, stubbornness or withdrawal from frustrating situations [12]. Difficulties recognizing negative facial expressions have also been documented [13].

WS results from a hemideletion of 26 genes on chromosome 7q11.23 and has a prevalence of 1 in 7,500 live births [14]. WS is characterized by mild to moderate ID or learning difficulties, dysmorphic facial features, heart disease (especially supravalvar aortic stenosis), connective tissue abnormalities, and failure to thrive or growth deficiency [15]. WS is associated with a relative strength in the structural and concrete components of language [16,17]. Individuals with WS are often described as demonstrating a distinct lack of inhibition with regard to approaching others in social contexts and as being gregarious and overly friendly [18,19]. Despite these seemingly positive characteristics, children with WS demonstrate significant difficulty interacting with others [20,21], high levels of anxiety [22,23], and significant impairments in pragmatic abilities [24,25].

Although progress has been made with regard to characterizing these behavioral phenotypes, much remains to be understood. To date, there are no published studies specifically designed to examine the social referencing process in individuals with either DS or WS. An evaluation of this process in DS and in WS that also includes an examination of the associated component abilities will allow for a better understanding of the difficulties encountered by these two groups of children in social interactions. Furthermore, it will provide insight into the roles that the component abilities play in the social referencing process in general.

To address these goals, we report four studies designed to begin to characterize the social referencing abilities of children with DS or WS. In the first study, we used a Social Referencing task to assess the regulatory function of social referencing. To obtain clarifying information as to where difficulties may be encountered, three additional studies were conducted that examined abilities fundamental to the social referencing process (initiating eye contact, gaze following and utilizing another person’s emotional reactions). Due to the rarity of these syndromes, the same groups of children with DS and children with WS participated in all four studies.

## Study 1: The regulatory function of social referencing

In the first study, we compared the behavioral responses of children with DS and children with WS on a standard social referencing task. Based on previous research, we predicted that children with DS would shift their attention more between the experimenter and the stimulus than would children with WS [26], even though children with WS would earn significantly higher standard scores both for overall intellectual ability and verbal ability on standardized measures of intellectual ability and receptive vocabulary [9,27]. We also predicted that children with WS would be more likely to produce ‘long looks’ to the experimenter [28] and to imitate the experimenter’s facial expressions than would children with DS [29].

### Method

#### Participants

The participants were recruited through an ongoing study of language and cognitive development of children with neurodevelopmental disorders at the University of Louisville Parental consent was obtained for all participants. There is no overlap between the participants included in the present project and those included in the study by John and Mervis [27].

The DS group included 21 children (13 boys, 8 girls) aged 3.51 to 5.88 years (mean = 4.97; SD = 0.74) with confirmed trisomy 21. The racial/ethnic background of this group was 90% White non-Hispanic and 10% biracial non-Hispanic (1 child African-American/White and 1 child Native-American/White).

The WS group included 21 children (13 boys, 8 girls) aged 3.52 to 5.94 years (mean = 4.92; SD = 0.76) with genetically confirmed classic-length WS deletions. The WS group was selected to match the DS group on chronological age (CA; *P* = 0.79) and gender from a larger pool of children with WS for whom social referencing data were obtained (n = 32). The racial constitution of the children in the final WS group was: 67% White, 5% African-American, 9% Asian, and 19% bi- or multiracial (1 child African-American/White, 2 children Pacific Islander/White, and 1 child African-American/Pacific Islander/White). None of the children was of Hispanic origin.

#### Standardized assessments

##### Differential Ability Scales–II Early Years (DAS-II-EY)

The DAS-II-EY [30] provides an assessment of general intellectual functioning for children aged 2½ to 8 years. In the present study we considered the general conceptual ability (GCA) standard score (similar to IQ) and the cluster standard scores (SSs) measuring verbal, nonverbal reasoning, and spatial abilities (mean = 100; SD = 15).

#### Procedure

Children completed a battery of cognitive and language assessments including an assessment of intellectual abilities (DAS-II-EY) and a measure of receptive vocabulary ability (PPVT-4). The DAS-II-EY and PPVT-4 were administered according to the standardized procedures and were almost always completed within a few days of the Social Referencing task.

##### Social referencing task [modeled after 1, 2, 3]

The Social Referencing task was designed to assess the child’s ability to regulate his/her own behavior toward an ambiguous stimulus in response to an adult’s behavioral reaction. Two trials (one Joyful trial and one Fearful trial) were administered to each child. The trials were conducted on different days and differed in terms of the stimulus, experimenter, experimenter’s behavioral reaction, and playroom, all of which were counterbalanced within each diagnostic group.

During the Social Referencing trials, the child and the experimenter played together on the floor of a familiar playroom that contained an ambiguous stimulus (remote-controlled robot covered by a cloth) placed on a child-height table located in the corner of the room. Once the child was positioned near the robot, an assistant activated the robot from behind a one-way mirror. To allow the child time to initiate eye contact with her, the experimenter waited either approximately 3 seconds or until the child made eye contact (whichever came first) before beginning her specified behavioral reaction. The experimenter’s behavioral reaction (“Signal stage”) lasted approximately 10 seconds. During the Signal stage, the experimenter alternated gaze between the stimulus and the child. Following the Signal stage, the experimenter entered the Neutral stage, during which she resumed looking at the toys on the floor to avoid giving the child further behavioral signals. During the Neutral stage, the experimenter refrained from interacting with the child unless the child became distressed or attempted to uncover the stimulus. Two cloth-covered robots were used as ambiguous stimuli; the robots varied in size, shape, movement pattern, motor sound, and color of cloth cover. The experimenters demonstrated facial expressions based on descriptions provided by Izard [32], and Ekman and Friesen [33], for the respective emotions.

#### Coding

Videotapes of the sessions were coded to examine the experimenter’s affective reaction and the child’s behavioral reaction. To assess reliability, a second person independently coded six randomly selected tapes, three in each diagnostic group, which were stratified by age (one 3-year-old, one 4-year-old, and one 5-year-old).

##### Experimenter affective reaction

To confirm that the intensity of the experimenter’s emotional display was similar across participants, the experimenter’s affective reaction during the Signal stage was coded from the videotape by a coder blind to the conditions and hypotheses of the study. The 3-step system for coding facial expressions developed by Hiatt *et al.*[34], which involved coding which emotions were present during the display, which emotion was the predominant emotion, and the intensity of the predominant emotion, was used. In addition to the target emotion, it was acceptable for the experimenter to demonstrate ‘surprise.’ For all participants, the intended target emotion was demonstrated by the experimenter and was rated to have an intensity rating of 4 or 5 on a 5-point scale for both trials. High reliability was observed (percentage of agreement = 96.43%, κ = 0.91) for which emotion(s) was/were present. High reliability was also observed for both predominant emotion and intensity of the predominant emotion (percentage of agreement = 100%, κ = 1.0).

##### Gazing behavior

Children’s gazing behavior was coded during the Ambiguous (interval prior to the onset of the experimenter’s reaction) and Signal stages of the task. A second coder, blind to the hypotheses of the study, assessed reliability. “Duration of longest look to the experimenter” and “rate of looks between the experimenter and stimulus” per minute during the Ambiguous and Signal stages of the task, were calculated separately for each condition. For both dependent variables, percentage of agreement (99%) and Cohen's kappa (κ = 0.99), using a tolerance window of 500 ms, were very high, indicating excellent reliability.

##### Acknowledgment of affective response/attention to experimenter and stimulus (acknowledged/attended)

This composite variable indicated whether or not children produced behaviors demonstrating that they both acknowledged the experimenter’s affective response (acknowledged) and paid attention to both the experimenter and the stimulus (attended). Children were coded as having ‘attended’ if they either shifted their gaze at least once from the experimenter to the stimulus during the experimenter’s display or produced verbalizations indicating that they understood the experimenter’s behavior was about the stimulus. In the Joyful condition, if the child smiled or produced a verbalization that referenced the experimenter’s affective state (e.g., “It is funny!”), he or she was coded as having ‘acknowledged.’ In the Fearful condition, if the child became distressed, tried to comfort the experimenter, or tried to explain to the experimenter why the stimulus was not scary, the child was coded as having ‘acknowledged.’ The child had to receive positive codes on both ‘attended’ and ‘acknowledged’ in order to be coded as ‘yes’ on the composite variable (acknowledged/attended). Percentage of agreement (91.67%) and Cohen's kappa (κ = 0.82) indicated high reliability.

##### Imitation of behavioral response

This variable represented situations in which the child imitated the experimenter’s behavioral response without demonstrating any other behavioral sign indicating that he or she was experiencing the response him- or herself. Percentage of agreement (91.67%) and Cohen's kappa (κ = 0.80) indicated high reliability.

##### Touch stimulus

This variable represented situations in which the child approached the stimulus and either touched it or was stopped by the experimenter just before he or she succeeded in touching it. Percentage of agreement (100%) and Cohen's kappa (κ = 1.0) indicated very high reliability.

##### Demonstrated a response regarding stimulus

This variable indicated whether or not the child demonstrated behaviors that communicated a response regarding the stimulus. Examples of behaviors indicating that the child demonstrated a response regarding the stimulus included: approach to stimulus, retreat from stimulus/distress, or generation of a hypothesis (verbally or through sign language) as to what the stimulus was (e.g., “It’s an elephant!”). If the child demonstrated a response regarding the stimulus, the coder also indicated whether the child's response was positive or negative. For example, approaching the stimulus with positive affect was coded as a positive response while retreating from the stimulus paired with communication of distress was coded as a negative response. Percentage of agreement (91.67%) and Cohen's kappa (κ = 0.83) indicated high reliability.

#### Data analysis

Standard scores from the DAS-II-EY and the PPVT-4 met the necessary statistical assumptions for use of parametric analyses. As the distributions for rate of looks between the experimenter and stimulus and duration of longest look violated the parametric assumptions of normality, Mann–Whitney U tests were conducted for analyses involving these measures. As the remaining dependent variables were dichotomous and did not violate the assumption of expected frequencies greater than 5, χ^2^ analyses were performed.

### Results

#### Intellectual abilities

Descriptive statistics for the DAS-II-EY GCA and cluster SSs and the PPVT-4 SSs and AE scores are presented in Table 1. As predicted, the mean GCA for the WS group was significantly higher than the mean GCA for the DS group [*t*(40) = 1.76, *P* = 0.045, Cohen’s *d* = −0.54, one-tailed test]. Follow-up *t*-tests were conducted on the DAS-II-EY cluster SSs to determine the locus of the significant effect (two-tailed α_fw_ = 0.017; one-tailed α_fw_ = 0.034). Also as predicted, the children with WS earned significantly higher Verbal SSs than did the children with DS, [*t*(40) = 2.53, *P* = 0.008, Cohen’s *d* = −0.78, one-tailed]. Comparisons for Nonverbal Reasoning SSs [*t*(40) = 1.90, *P* = 0.06, Cohen’s *d* = −0.59, two-tailed] and Spatial SSs [*t*(40) = 0.39, *P* = 0.70, Cohen’s *d* = −0.06, two-tailed] did not reach criterion for a significant difference between groups. Finally, as predicted, the children with WS earned significantly higher PPVT-4 SSs than did the children with DS, [*t*(40) = −2.60, *P* = 0.01, Cohen’s *d* = −0.82, one-tailed].

**Table 1:** Descriptive statistics for performance on the DAS–II–EY and PPVT–4 as a function of syndrome

	**Down syndrome**			**Williams syndrome**		
**Measure**	**Mean**	**SD**	**Range**	**Mean**	**SD**	**Range**
**DAS–II–EY**						
Verbal cluster SS	68.00	10.72	51 – 91	76.90	12.05	51 – 95
Nonverbal reasoning cluster SS	76.95	14.13	36 – 97	84.38	10.97	63 – 107
Spatial cluster SS	51.19	11.72	34 – 70	52.76	14.05	34 – 82
GCA	59.19	10.49	37 – 73	65.05	11.03	44 – 81
**PPVT–4**						
SS	79.55	10.63	61 – 101	88.65	11.50	66 – 106
AE–score (in years)	3.42^+^	–	2.08 – 5.33	3.87^+^	–	2.58 – 6.42

^+^Median.

#### The regulatory function of social referencing

One girl with DS was excluded from the present set of analyses due to experimenter deviation from the administration procedures. To keep the number of participants in each group the same, the data for the girl with WS closest in age to the excluded participant with DS were also excluded. No significant effect of order was observed on any of the dependent variables (all *P* > 0.20). Therefore, order was not included as a variable in any of the subsequent analyses.

##### Rate of looking between experimenter and stimulus

Descriptive statistics for the gazing behavior variables are presented in Table 2. As predicted, the distributions of rate of looks between the experimenter and the stimulus differed significantly (α_fw_ = 0.025, one-tailed) for the two diagnostic groups in both conditions, with children with DS demonstrating higher rates of looking between the experimenter and the stimulus than did children with WS (Joyful: *U* = 89.00, *P* = 0.002, *r* = −0.48; Fearful: *U* = 121.00, *P* = 0.02, *r* = −0.34).

**Table 2:** Descriptive statistics for performance on gazing behavior variables as a function of condition

	**Down syndrome**		**Williams syndrome**	
**Measure**	**Median**	**Range**	**Median**	**Range**
	Joyful condition		Joyful condition	
Rate of looks^+^	10.67	3.28 – 19.89	6.72	0.00 – 18.86
Duration of longest look (in sec.)	2.25	0.58 – 6.62	4.13	0.68 – 13.17
	Fearful condition		Fearful condition	
Rate of looks^+^	9.98	3.14 – 18.00	7.54	0.00 – 13.36
Duration of longest look (in sec.)	2.68	1.23 – 11.54	4.33	0.91 – 8.67

^+^Number of looks between experimenter and stimulus per minute.

##### Duration of longest look

In the Joyful condition, the distributions of duration of longest look produced by the child did not reach criterion for a significant difference (α_fw_ = 0.025, one-tailed test) between diagnostic groups (*U* = 222.00, *P* = 0.11, *r* = 0.20). However, as predicted, the duration of the longest look was significantly longer for children with WS than for children with DS in the Fearful condition (*U* = 218.00, *P* = 0.01, *r* = 0.35).

##### Acknowledgment of affective response/attention to examiner and stimulus (Acknowledged/Attended)

In the Joyful condition, significantly more (α_fw_ = 0.025; Table 3) children with DS than children with WS were coded as having acknowledged/attended [χ^2^(1) = 5.01, *P* = 0.025, odds ratio = 4.50, *CI*_*.95*_ = 1.17, 17.37]. The five children with DS who were coded as not having acknowledged/attended were coded as having attended to both the experimenter and stimulus during the experimenter’s display but were not coded as having acknowledged the experimenter’s affective response. Of the 12 children with WS who were not coded as having acknowledged/attended, 3 were coded as having neither acknowledged nor attended and 9 were coded as having attended but not as having acknowledged the experimenter’s affective response.

**Table 3:** Number of children who demonstrated various coded behaviors during the Social Referencing task as a function of condition

**Coded behavior**	**Down syndrome (n = 20)**	**Williams syndrome (n = 20)**
	Joyful condition	Joyful condition
Acknowledged/Attended	15	8
Imitated experimenter	1	4
Touched stimulus*	16	10
Demonstrated a response	16	13
	Fearful condition	Fearful condition
Acknowledged/Attended	12	7
Imitated experimenter	2	8
Touched stimulus*	10^+^	3
Demonstrated a response	13	5

^*^Includes children who attempted to touch the stimulus but were stopped by the experimenter.

^+^Includes one child who picked up the stimulus and threw it across the room.

The relation between diagnostic group and acknowledged/attended did not reach criterion for a significant association in the Fearful condition [χ^2^(1) = 2.51, *P* = 0.11, odds ratio = 2.79, *CI*_*.95*_ = 0.77, 10.04]. All nine children with DS who were coded as not having acknowledged/attended were coded as having attended to both the experimenter and stimulus during the experimenter’s display but were not coded as having acknowledged the experimenter’s affective response. Of the 13 children with WS who were coded as not having acknowledged/attended, 1 child was coded as have neither acknowledged nor attended and 12 were coded as having attended but not as having acknowledged the experimenter’s affective response.

##### Imitation of behavioral reaction

The relation between diagnostic group and imitation did not meet criterion for a significant association (α_fw_ = 0.025; Table 3) in the Joyful condition [χ^2^(1) = 2.06, *P* = 0.08, odds ratio = 0.21, *CI*_*.95*_ = 0.02, 2.08, one-tailed test]. In the Fearful condition, as predicted, children with WS were more likely than were children with DS to imitate the experimenter’s behavioral response [χ^2^(1) = 4.80, *P* = 0.02, odds ratio = 6.01, *CI*_*.95*_ = 1.08, 33.28, one-tailed test].

##### Touch stimulus

The relation between diagnostic group and touching the stimulus was not significant (α_fw_ = 0.025; Table 3) in the Joyful condition (χ^2^(1) = 3.96, *P* = 0.047, odds ratio = 4.0, *CI*_*.95*_ = 0.98, 16.27). However, significantly more children with DS than children with WS touched the stimulus in the Fearful condition [χ^2^(1) = 5.58, *P* = 0.02, odds ratio = 5.67, *CI*_*.95*_ = 1.25, 25.61].

##### Demonstrated a response regarding stimulus

The relation between diagnostic group and whether or not the child demonstrated a response regarding the stimulus (α_fw_ = 0.025; Table 3) did not reach criterion for a significant association in the Joyful condition [χ^2^(1) = 1.13, *P* = 0.29, odds ratio = 2.15, *CI*_*.95*_ = 0.52, 9.00]. Most children with DS (15/16) and most children with WS (11/13) who were rated to have demonstrated a response regarding the stimulus were rated to have demonstrated a positive response.

In contrast, significantly more children with DS than children with WS demonstrated a response regarding the stimulus in the Fearful condition, [χ^2^(1) = 6.47, *P* = 0.01, odds ratio = 5.57, *CI*_*.95*_ = 1.42, 21.86]. Despite the experimenter's fearful reaction, only a small proportion of the children who demonstrated a response regarding the stimulus in the Fearful condition (5/13 children with DS and 2/5 children with WS) were rated as having demonstrated a negative response. Of the 8 children with DS who demonstrated a positive response, 5 saw the experimenter's fearful reaction and then approached the stimulus, 1 watched the experimenter's fearful reaction for its entire duration and slyly approached the stimulus while checking to make sure the experimenter was not looking, 1 stated the stimulus was an elephant and maintained this position even after seeing the experimenter’s fearful reaction, and 1 communicated to the experimenter that she should not be afraid and approached the stimulus to show her it was okay. The 3 children with WS who demonstrated a positive response regarding the stimulus saw the experimenter’s fearful reaction and then approached the stimulus.

### Discussion

The results of Study 1 indicated differences in the behavioral responses of children with DS and children with WS both in situations in which the experimenter communicated a joyful message and in situations in which the experimenter communicated a fearful message about an ambiguous stimulus. When the experimenter communicated a joyful message, although the majority of children in both groups demonstrated positive responses regarding the stimulus, between group differences were observed in the children’s behavioral responses. The children with DS not only shifted their attention between the adult and the stimulus more than did the children with WS, but they also were more likely to produce behaviors acknowledging the experimenter’s joyful message while attending to both the experimenter and the stimulus. As such, even though the majority of children in both groups demonstrated positive responses regarding the stimulus when the experimenter’s response was positive, the children with DS demonstrated more behaviors than did the children with WS indicating that they indeed saw the experimenter’s message and were simultaneously attending to the stimulus.

When the experimenter communicated a fearful message about an ambiguous stimulus, only 38% of the children with DS and 40% of the children with WS who demonstrated a response regarding the stimulus demonstrated a negative response, despite all the children clearly seeing the experimenter’s fearful reaction. Interestingly, once again between-group differences were observed in the children’s behavioral responses. The children with DS shifted their attention between the experimenter and the stimulus more frequently than did the children with WS and were more likely to touch the stimulus. For children with WS, the length of the longest look to the experimenter was considerably longer than it was for children with DS. In addition, the children with WS were more likely to imitate superficially the experimenter’s fearful reaction than were the children with DS.

So, do children with DS or children with WS use the reactions of another person to guide their own behavior in ambiguous situations? This question is difficult to answer from these data alone, as it remains unclear why these differences occurred and what they indicate. Within this naturalistic interaction, the components are so tightly intertwined that it is difficult to pinpoint the exact point(s) where a problem may have occurred. To obtain insight into potential area(s) of difficulty, in the three remaining studies we examined children’s performance on tasks designed to press for each of the abilities that are fundamental to the social referencing process.

## Study 2: Initiating eye contact

To succeed within the social referencing process, the child must be able to coordinate his or her attention between the ambiguous stimulus and the experimenter. The ability to initiate eye contact is important to the social referencing process, as the child must shift his or her attention from the stimulus to the experimenter in order to gain access to the message (experimenter's reaction). This ability has been argued to serve as a precursor for the later development of understanding other people’s intentions, feelings and thoughts [35,36]. TD infants begin to coordinate attention between people and objects between 8 and 10 months of age [37,38], and between 12 and 15 months of age coordinated attention is consistently observed in triadic interactions [39].

Although social interaction skills are considered an area of relative strength for children with DS, difficulties pertaining to initiating eye contact have been documented. In situations in which both adult communicative partners and toys are available, toddlers with DS tend to maintain eye contact with the communicative partner or engage solely with the communicative partner much longer than do mental age (MA) matched and receptive language-age (RLA) matched TD children or children with autism [40-43]. In addition, toddlers and preschoolers with DS are significantly less likely to make triadic requests than are MA-matched TD children [8,44] or MA-matched children with mixed or non-specific etiology developmental delay (DD) [8].

There have been only a few studies pertaining to initiating eye contact in children with WS. Children with WS have been found to engage solely with another person (rather than attending to both a person and an object) significantly more than MA-matched TD children [45]. Children with WS also are significantly less likely to initiate requests and demonstrate a trend to share awareness less often (*P* = 0.07) than do MA-matched TD children [45].

Only one study has directly compared children with DS to children with WS. Using a standardized play-based assessment, Rowe *et al.*[26] compared toddlers with WS [mean(CA) = 26.6 months] to toddlers with DS matched on CA, developmental quotient (DQ), and expressive vocabulary size. Results indicated that the children with WS were significantly less likely to share awareness or shift gaze than were the children with DS. The two groups did not differ on frequency of triadic requests produced.

In order to examine children's ability to initiate eye contact with an adult, an important ability involved in social referencing, in Study 2 we investigated the looking behavior of preschoolers with DS or WS in three situations in which looking at the experimenter would require the child to shift his or her attention away from a toy of interest. Based on previous research [26,45], we predicted that children with DS would be more likely to spontaneously look to the experimenter in triadic interactions than would children with WS.

### Method

#### Participants

Participants were the 42 children described in Study 1. One boy with DS consistently cried in response to the Teasing condition. For this reason, his data, as well as those of the boy with WS closest in age to him, were excluded from the Teasing condition analyses.

#### Procedures

The children were administered a task developed by Phillips *et al.*[46]. This task, which will be referred to as the Initiating Eye Contact task, was designed to assess the child’s use of gaze in response to adult actions that varied with regard to the ambiguity of the adult’s intention. The task trials were administered during two structured play interactions at a table, each with a different experimenter and on a different day. The experimenter sat across the table from the child while they played together with a series of developmentally appropriate toys, one toy at a time. In the context of a play activity, the experimenter encouraged the child to play with the toy. Once the child was interested, the experimenter offered the toy to the child. Two of the three actions (blocking and teasing) produced by the experimenter were ambiguous, creating a situation that would solicit the child’s attention. The intention behind the experimenter's third action (give) was not ambiguous and therefore allowed the opportunity to evaluate the child’s spontaneous (unsolicited) initiation of eye contact.

Six trials per condition were administered (three trials with one experimenter on Day 1 and three trials with the second experimenter on Day 2). Order of administration was counterbalanced as described by John [47]. Administration procedures for the three conditions were as follows:

· Blocking: The experimenter handed the toy to the child. When the child was engaged with the toy both manually and visually, the experimenter covered the child’s hands with her own hands, preventing the child from further activity. This action was held for 4 seconds while looking at the child with neutral affect or until the child initiated eye contact (whichever came first), at which point the experimenter removed her hands.

· Teasing: The experimenter offered the toy to the child. As the child reached for the toy, the experimenter quickly withdrew it and held the toy out of the child’s reach for 4 seconds while looking at the child with neutral affect. After 4 seconds had elapsed or the child initiated eye contact (whichever came first), the child was given the toy.

· Giving: The experimenter handed the toy to the child while looking at him or her with neutral affect and the child was allowed to play with it.

#### Coding

The primary coder watched the session videotapes and coded whether or not the child made eye contact with the experimenter within 4 seconds of the start of the trial using The Observer XT 10.0 [48]. The start of the trial was defined as follows: i) Blocking condition: when the experimenter placed her hands over the child’s hands; ii) Teasing condition: when the experimenter pulled the toy away from the child; iii) Giving condition: when the child placed his or her hand on the toy. A second person independently coded three videotapes for each diagnostic group, (one 3-year-old, one 4-year-old, and one 5-year-old) to assess reliability. Percentage of agreement (92.59%) and Cohen's kappa (κ = 0.85) indicated high reliability.

#### Data analysis

Data from this task violated the parametric assumption of normality. As such, Wilcoxon signed-rank test and Mann–Whitney U test statistics were computed.

### Results

Due to administration errors, problems with videotape quality, or child refusal, data for 9/240 trials in the Blocking condition and 14/240 trials in the Teasing condition could not be scored. Accordingly, performance was measured by proportion of trials on which eye contact was made within 4 seconds of the start of the trial. Descriptive statistics for each condition are reported in Table 4.

**Table 4:** Descriptive statistics for proportion of trials on which children looked at the experimenter's face within four seconds of trial onset in the Initiating Eye Contact task

		**Down syndrome**		**Williams syndrome**	
**Measure**	**Action type**	**Median**	**Range**	**Median**	**Range**
Blocking	Ambiguous	0.80	0.17 – 1.00	0.83	0.00 – 1.00
Teasing	Ambiguous	0.67	0.17 – 1.00	0.83	0.00 – 1.00
Giving	Non–ambiguous	0.33	0.00 – 1.00	0.33	0.00 – 0.67

#### Within-group comparisons across conditions

Results indicated that the distributions of performance on the two ambiguous action conditions (Block and Tease) did not reach criterion for a statistical difference for either children with DS (*T* = 92.50, *P* = 0.20, *r* = 0.29) or children with WS (*T* = 63.00, *P* = 0.79, *r* = −0.06). Analyses comparing performance on the Blocking condition to performance on the Giving condition indicated that both children with DS (*T* = 15.00, *P* = 0.003, *r* = −0.65) and children with WS (*T* = 7.00, *P* < 0.001, *r* = −0.80) looked at the experimenter on a larger proportion of the trials in the Blocking condition than in the Giving condition (α_fw_ = 0.025). Similarly, results indicated that both children with DS (*T* = 3.00, *P* < 0.001, *r* = −0.81) and children with WS (*T* = 5.00, *P* < 0.001, *r* = −0.82) looked at the experimenter on a larger proportion of the trials in the Teasing condition than in the Giving condition.

#### Between-group comparisons across conditions

The distributions for the proportion of trials on which the child made eye contact in the Blocking (*U* = 207.00, *P* = 0.73, *r* = −0.05) and Teasing (*U* = 200.50, *P* = 0.99, *r* = 0.002) conditions did not reach criterion for a significant difference between groups (α_*fw*_ = 0.025). As predicted, the distributions for the proportion of trials on which the child made eye contact in the Giving condition differed significantly between the two groups (*U* = 304.00, *P* = 0.02, *r* = −0.33, one-tailed), with children with DS looking at the experimenter on a larger proportion of the Giving trials than did children with WS.

### Discussion

In Study 2, we considered the likelihood that children would shift attention from an object to an adult in response to the adult's production of ambiguous (solicited attention) and non-ambiguous (non-solicited attention) actions. Results indicated that both children with DS and children with WS were more likely to look at the experimenter in response to her ambiguous actions (blocking or teasing) than they were to look at her in response to her unambiguous action (giving). In addition, the two groups of children were similar in terms of their likelihood of looking at the experimenter in response to her ambiguous actions; most children in both groups looked at the experimenter in these situations. These results demonstrate that both groups of children are likely to look to the adult if she produces an action that prevents the child from gaining access to an object of interest. By preventing the child from accessing the object, the experimenter produced an action that solicited the child’s attention. The findings from the present study suggest that both children with DS and children with WS use eye contact relatively well in situations in which an adult’s ambiguous gesture solicits the child’s attention. It is important to note that it is also possible that, because the adult controls access to the object when she produces the ambiguous situation, the interaction was changed from a triadic interaction to a dyadic interaction. The existing literature on gazing behaviors demonstrates that both children with DS and children with WS use eye contact relatively well in dyadic interactions [42,45].

The findings from Study 2 also demonstrated, as predicted, that children with DS were more likely to look to the experimenter in response to her giving action than were the children with WS. Since the experimenter's giving action did not attempt to draw the child's attention to her, this situation is a triadic interaction in which eye contact by the child would indicate a spontaneous initiation of joint attention. As such, this finding is consistent with prior literature demonstrating that children with DS are more likely to initiate joint attention in triadic situations than are children with WS of the same CA [26].

When one considers the social referencing process, the child not only has to look at the adult, he/she must also be able to identify the focus of the adult’s attention. That is, the child must be able to follow the adult’s gaze to determine the referent of interest. In Study 3, we evaluated children's ability to follow another person's gaze, the fundamental ability involved in linking the experimenter's message to an object.

## Study 3: Gaze following

The ability to follow another person’s gaze is an important milestone in early development as it is a governing factor in both social and communicative interactions between a child and an adult [49,50]. So, what drives a person to look in the same direction as another person? Most basically, it is theorized to be the desire to see what the other person is looking at, or the desire to share in another person’s perspective [51]. Although this is likely what compels adults to follow gaze, this may not be the case throughout development.

While some theorists have suggested that the ability of young children to follow another person’s gaze indicates the understanding of intentionality [35,52], other researchers have strongly cautioned against this interpretation, pointing out that children must be sensitive to changes in eye direction specifically in order to demonstrate an understanding of joint visual attention [51]. Whereas prior to age 10 months TD infants will turn their heads in response to another person’s change in head direction, it is only around 10 months of age that they will selectively follow changes in eye gaze specifically [53,54].

Only a few studies have specifically examined gaze following by children with DS. Results of these studies indicate that preschool and school-aged children with DS perform similarly to TD children and children with DD matched on RLA and significantly better than children with autism matched on RLA [55,56]. Two studies report specific information regarding the proportion of older toddlers and preschoolers with WS who followed a gazing gesture paired with a verbalization during a standardized play based assessment. Klein-Tasman and colleagues [57] found that 66% of the children in their sample were able to follow an examiner’s communicative gaze shift paired with the verbalization “Look.” Lincoln *et al.*[58] reported that 80% of their sample could follow a communicative eye-gaze gesture accompanied with “Look.”

To date only one study has directly compared the gaze following behavior of children with DS and children with WS. John and Mervis [27] examined the comprehension of the communicative intent behind pointing and gazing gestures by preschool-age children with DS and children with WS. Results indicated that despite significantly lower DQs and language raw scores, children with DS were significantly better at comprehending communicative intent. Both groups were better at comprehending communicative intent indicated by a pointing gesture paired with eye gaze than by eye gaze alone. Performance between the two groups was not directly compared on the gaze following condition alone. As discussed by John and Mervis [27], the children with DS performed most similarly to the TD 18-month-olds reported by Behne and colleagues [59] whereas the children with WS performed most similarly to the TD 14-month-olds.

In the present study, in order to examine the ability to follow another person’s gaze, we investigated the likelihood that children with DS or WS would follow another person's head turn when her eyes were open *versus* when her eyes were closed. Based on a previous study [27], it was predicted that children with DS would be more likely than would children with WS to look in the same direction as an adult in the Eyes Open condition.

### Method

#### Participants

Participants were the 42 children described in Study 1.

#### Procedure

The children were administered a Gaze Following task [54,60] which was designed to provide information as to whether following an adult’s gaze reflects an understanding of the gazer’s focus of attention; that is, the child only turns his/her head in the same direction as the adult when the adult’s eyes are open. Six trials in each condition were administered (three trials with one experimenter on Day 1 and three trials with a different experimenter on Day 2).

The experimenter and child played at a table with a series of developmentally appropriate toys. Before the start of each trial, the experimenter removed the toy with which the child had been playing from view. Next, after establishing eye contact with the child, the experimenter turned her head toward a target (left or right) while demonstrating the appropriate cue paired with a subtle vocalization (“Oh, hmm”). The experimenter demonstrated one of two cues:

· Eyes Open: The experimenter turned her head and eyes toward the object with eyes open and said “Oh, hmm.” The experimenter fixated on the target for 5 seconds and then returned to midline, made eye contact with the child, and resumed the play interaction.

· Eyes Closed: The experimenter established eye contact with the child, closed her eyes, and turned her head toward the object and said “Oh, hmm.” The experimenter maintained this position for 5 seconds and then returned to midline, opened her eyes, and resumed the play interaction.

Order of condition and target location was determined quasi-randomly as described in John [47].

#### Coding

A primary coder, blind to the condition and direction of the experimenter’s head turn, coded all of the session videotapes. The targets were not visible in the video. However, the coder was informed that targets were located on the walls to both the left and the right between the child and the experimenter. The coder scored each trial for whether or not the child intentionally looked for a target and, if so, indicated the direction of the child’s look.

After all of the trials were coded, the coder was informed as to the location of the target and recorded if the target the child looked at was the same target the experimenter turned her head toward. A variable (“follow”) was computed indicating the number of trials during which the child looked at the correct target within 5 seconds of the experimenter's head turn, separately for each condition. A second person independently coded three randomly selected tapes for each diagnostic group (one 3-year-old, one 4-year-old, and one 5-year-old). Percentage of agreement (97.8%) and Cohen's kappa (κ = 0.96) indicated very high reliability.

#### Data analysis

Data from this task violated the parametric assumption of normality. As such, Wilcoxon signed-rank test and Mann–Whitney U test statistics were computed.

### Results

Analyses were computed to determine if order of condition and direction of first trial affected children’s following performance. Analyses indicated no significant effect of condition (*P* = 0.77) or direction of first trial (*P* = 0.81) on the dependent variable. Therefore, order was not included as a variable in any of the subsequent analyses.

Descriptive statistics for performance in the two conditions are reported in Table 5. Results indicated that the distributions of gaze following differed significantly as a function of condition for both children with DS (*T* = 2.00, *P* < 0.001, *r* = 0.87) and children with WS (*T* = 4.00, *P* = 0.001, *r* = 0.75), with children in both groups significantly more likely to follow the experimenter's head turn in the Eyes Open condition than in the Eyes Closed condition.

**Table 5:** Descriptive statistics for 'Following' as a function of diagnostic group in the Gaze Following task

	**Down syndrome**		**Williams syndrome**	
**Measure**	**Median**	**Range**	**Median**	**Range**
Eyes open follow	5.00	1.00 – 6.00	4.00	0.00 – 6.00
Eyes closed follow	1.00	0.00 – 4.00	0.00	0.00 – 3.00

The distributions of performance in the Eyes Closed condition did not differ significantly between the two groups (*U* = 270.00, *P* = 0.16, *r* = 0.22). In contrast, as predicted, the distributions of performance in the Eyes Open condition differed significantly between the two diagnostic groups (*U* = 300.00, *P* = 0.025, *r* = 0.32, one-tailed test) with children with DS more likely than children with WS to follow the experimenter's head turn in the Eyes Open condition.

### Discussion

In Study 3, we considered children's ability to follow another person's gaze. Results indicated that both groups of children demonstrated sensitivity to the communicative meaningfulness of a person's eyes. At the same time, children with DS were more likely to follow another person's head turn paired with open eyes than were children with WS. This finding adds more evidence to the growing body of literature demonstrating that children with WS evidence impairments in responding to joint attention [26,27,45,57].

Thus far, we have presented data concerning two abilities that are fundamental to the social referencing process: initiating eye contact and following another person’s gaze. In the fourth study, we examined the final ability fundamental to the social referencing process, the child's ability to recognize the communicative significance of another person's affective reaction.

## Study 4: Utilizing emotional reactions

Emotional reactions are more than an index of underlying states; they also have an interpersonal function, regulating the behavior of other individuals. It is this function of emotion that makes affective expression an important component of the communicative process [61]. By 6 months of age, TD infants respond differentially to their mothers’ happy and sad expressions [62,63], and by 12 months of age TD infants use emotional information communicated by another person to help appraise an ambiguous situation [3,5].

Only a few studies of emotion recognition by children with DS have been conducted. Kasari *et al.*[64] found that children with DS [mean(CA) = 6.39 years] performed significantly worse than CA-matched TD children but similarly to MA-matched TD children [mean(CA) = 3.30 years] on both emotion labeling and emotion recognition tasks. The children in all three groups were more accurate at identifying, recognizing and labeling happy expressions than fearful expressions. In a second study, the authors found that children with DS [mean(CA) = 8.13 years] were significantly worse at identifying fear within a story scenario than were MA-, RLA- and expressive language age (ELA) matched TD children and CA-, MA-, RLA- and ELA-matched children with DD of mixed etiology. When children with DS incorrectly identified the emotion 'fear,' their responses were significantly more likely to be that of positively valenced emotions than were the incorrect responses of children with DD or TD children. Williams e*t al.*[65] found that an older group of children with DS [mean(CA) = 13.33 years] performed similarly to MA-matched children with non-specific ID [mean(CA) = 11.83 years] but significantly worse than MA-matched TD children on matching expressions of fear [mean(CA) = 3.63 years].

Children with WS also evidence difficulty matching and labeling emotional expressions. Tager-Flusberg and Sullivan [66] found that children with WS [mean(CA) = 7.17 years] evidenced impairments on a task involving matching facial expressions that were comparable to children with Prader-Willi syndrome [mean(CA) = 6.92 years] and children with non-specific ID [mean(CA) = 7.58 years] matched on CA, IQ and language ability. Relative to TD children, Gagliardi *et al.*[67] found that individuals with WS [mean(CA) = 14.35 years] performed significantly worse than CA-matched TD individuals but similarly to MA-matched TD children [mean(CA) = 5.5 years], on identifying animated expressions of both happiness and fear. Individuals in all three groups were significantly better at recognizing happy expressions than fearful expressions.

Porter [13] conducted the only study which directly compared the ability of individuals with DS and individuals with WS to recognize emotions on a standardized assessment. Results indicated that, when controlling for MA, individuals with WS [range(CA): 6.0 to 43.67 years] and individuals with DS [range(CA): 6.75 to 40.75 years] demonstrated comparable performance with regard to recognizing expressions of fear. High mean proportions of errors for expressions of fear were observed for both groups, even when high intensity stimuli were used. Results of these studies suggest that both individuals with DS and individuals with WS have more difficulty recognizing fearful expressions than do their same age TD peers. Overall, however, very little is known about emotion recognition in individuals with DS or WS, especially in young children.

In the present study, we evaluated whether or not the behavioral responses of children with DS and children with WS differed on a task similar to the Social Referencing task (Study 1) with one key difference, a reduction in the attentional demands on the child. Based on previous research [29], it was predicted that children with WS would be more likely to superficially imitate the experimenter’s behavioral response than would children with DS.

### Method

#### Participants

Participants were the same sample of children reported for the Social Referencing task analyses in Study 1. The present study used a between-subjects design with approximately half of the children in the Joyful condition and half of the children in the Fearful condition. (See the Procedure section for an explanation of why a between-subjects design was used). The sample of children in the Joyful condition included 11 children with DS (8 boys, 3 girls) aged 3.77 to 5.87 years (mean = 4.88, SD = 0.79) and 11 children with WS (6 boys, 5 girls) aged 3.52 to 5.81 years (mean = 4.80, SD = 0.83). The sample of children in the Fearful condition included 9 children with DS (5 boys, 4 girls) aged 3.51 to 5.88 years (mean = 5.07, SD = 0.75) and 9 children with WS (7 boys, 2 girls) aged 3.70 to 5.94 years (mean = 5.07, SD = 0.73). The two diagnostic groups were well matched for CA in both the Joyful condition (*P* = 0.67) and the Fearful condition (*P* = 0.97).

#### Procedure

The Surprise Box task, modeled after the task described by Scambler *et al.*[68], was designed both to evaluate children’s responses to other people’s emotional reactions (Joyful and Fearful) and to elicit emotional responses from children. While the Surprise Box task is similar to the Social Referencing task used in Study 1, there are two key differences: i) In the Surprise Box task the “referent” is in a box held by the experimenter, making it easier for the child to shift attention from the “referee” to the “referent;” and ii) in the Surprise Box task the experimenter assumes the responsibility of ensuring she has the child's attention prior to demonstrating her behavioral reaction toward the contents of the box.

Each child participated in six trials, three on Day 1 and three on Day 2. Only the first of each participant's six trials was included in the analyses, resulting in a between-subjects design. This decision was made based on experimenter observations that the children clearly demonstrated carryover from the previously administered trials, which were verified by statistical analyses that indicated significant order effects (*P* = 0.02).

The box was paired with one of two behavioral reactions communicated by the experimenter (Joyful or Fearful). In the context of a play activity, the experimenter pulled out a small gift box and said, “I wonder what’s in here.” At no point during the trial was the child able to see the contents of the box. Once the experimenter had the child’s attention, she opened the box and, while looking inside, demonstrated the specified behavioral reaction, following the same procedures outlined in Study 1. Playroom location, experimenter and behavioral reaction were alternated across participants in each diagnostic group.

#### Conditions

The experimenters demonstrated the facial expressions of joy and fear using the procedures outlined in Study 1.

#### Coding

The primary coder, blind to the hypotheses of the study, coded all of the session videotapes. To assess reliability, a second person independently coded three randomly selected tapes for each diagnostic group (one 3-year-old, one 4-year-old, and one 5-year-old). The procedures described in Study 1 were used to code five variables: experimenter affective display, acknowledgement of behavioral response and attending to both experimenter and stimulus, imitation of behavioral response, reach for box, and demonstration of response regarding stimulus. For each variable, percentage of agreement was 100% and Cohen's kappa was 1.0.

#### Data analysis

As all of the dependent variables were dichotomous and the assumption of expected frequencies greater than 5 necessary to perform a χ^2^ analysis was violated, Fisher-exact test statistics were computed.

### Results

Descriptive information regarding the proportion of children who demonstrated the behaviors coded is presented in Table 6.

**Table 6:** Proportion of children who demonstrated various coded behaviors during the Surprise Box task as a function of diagnostic group

**Coded behavior**	**Down syndrome**	**Williams syndrome**
	Joyful condition	Joyful condition
Acknowledged/Attended	8/11	7/11
Imitated	0/11	0/11
Touched	10/11	11/11
Demonstrated a response	9/11	11/11
	Fearful condition	Fearful condition
Acknowledged/Attended	2/9	3/9
Imitated	0/9	0/9
Touched	6/9	6/9
Demonstrated a response	6/9	5/9

#### Acknowledgment of affective response and attention to experimenter and stimulus (Acknowledged/Attended)

As indicated in Table 6, the majority of children in both diagnostic groups were coded as having acknowledged/attended in the Joyful condition, but in the Fearful condition few children did so. The relation between these variables was not significant (α_fw_ = 0.025) in either the Joyful [χ^2^(1, n = 22) = 0.21, Fisher’s exact *P* = 1.00, odds ratio = 1.52, *CI*_*.95*_ = 0.11, 4.00] or the Fearful [χ^2^(1, n = 18) = 0.28, Fisher’s exact *P* = 1.00, odds ratio = 0.57, *CI*_*.95*_ = 0.07, 4.64] conditions.

#### Imitation of behavioral reaction

None of the children imitated the experimenter’s behavioral reaction.

#### Reach for box

The relation between diagnostic group and whether or not the child reached for the box was not significant (α_fw_ = 0.025) in either the Joyful condition [χ^2^(1, n = 22) = 1.05, Fisher’s exact *P* = 1.00, odds ratio could not be computed as 1 cell had a frequency of 0] or the Fearful condition [χ^2^(1, n = 18) = 1.05, Fisher’s exact *P* = 1.00, odds ratio = 1.00, *CI*_*.95*_ = 0.19, 5.36].

#### Demonstration of response regarding stimulus

The relation between diagnostic group and demonstration of response regarding stimulus did not reach criterion for a significant association in the Joyful condition [χ^2^(1, n = 22) = 2.22, Fisher’s exact *P* = 0.48, odds ratio could not be computed as 1 cell had a frequency of 0]. All of the children in both diagnostic groups who were rated as having demonstrated a response demonstrated a positive response regarding the contents of the box. The relation between these variables also did not reach criterion for a significant association for the Fearful condition [χ^2^(1, n = 18) = 0.23, Fisher’s exact *P* = 1.00, odds ratio = 1.60, *CI*_*.95*_ = 0.24, 10.81]. Of the children who were rated as having demonstrated a response regarding the contents of the box, only one child with DS (of 6) and one child with WS (of 5) was rated as having demonstrated a negative response. Of the 5 children with DS who demonstrated a positive response, 4 saw the experimenter's fearful reaction and then reached for the box and 1 decided the stimulus was an owl and maintained this position even after seeing the experimenter’s fearful reaction. For the 4 children with WS, 2 saw the experimenter's fearful reaction and then reached for the box, 1 communicated to the experimenter that she should not be afraid and intensely laughed, and 1 demonstrated concerned intonation while asking what was wrong and reaching for the box and then quickly changed to positive intonation while he asked if he could see what was inside.

### Discussion

In Study 4, we considered children’s utilizations of another person’s emotionally valenced communications in a task with a reduced attentional demand on the child. When the experimenter communicated a joyful message about the ambiguous stimulus, results indicated that the reactions of children with DS and children with WS were comparable. The majority of children in both groups produced behaviors acknowledging the experimenter's joyful message and demonstrating that they attended to both the experimenter and the stimulus. In addition most children in both groups demonstrated a positive response regarding the contents of the box (DS = 82%, WS = 100%).

When the experimenter communicated a fearful message about the ambiguous stimulus, the reactions of the two groups were comparable to each other and differed from the pattern of findings for the Joy condition. Few children in either group produced behaviors that demonstrated that they acknowledged the experimenter's fearful reaction and attended to both the experimenter and the stimulus. In fact, the majority of children in both groups reached for the stimulus. Finally, although the majority of children with DS (67%) and children with WS (56%) demonstrated a response regarding the fearful stimulus, only one child in each group demonstrated a negative response. In addition, despite clearly seeing the experimenter's fearful expression, most children who demonstrated a positive response regarding the contents of the box reached for the box without communicating disagreement with the experimenter.

While one must be careful in interpreting the findings in Study 4 due to the limited sample size, the reactions of children in the two groups were quite similar. These findings, indicating that a much higher proportion of children in both groups demonstrated a response consistent with the adult’s emotional reaction in the Joyful condition than in the Fear condition, are consistent with prior findings that both children with DS and children with WS more accurately interpret happy facial expressions than fearful facial expressions [13,64,65,67,69].

## General discussion

The General Discussion is organized as follows: We i) briefly summarize and discuss the project findings; ii) present an integrative picture of social referencing as a function of diagnostic group; and, iii) discuss the implications of these findings for the development of the behavioral phenotypes of these syndromes.

### Summary of findings

On the Social Referencing task (Study 1), clear differences were observed in children’s behavioral responses both as a function of the experimenter’s emotional display and as a function of diagnostic group. In the Joyful condition, the majority of children in both groups demonstrated responses regarding the stimulus that were consistent with the adult’s response; this was not the case for either diagnostic group in the Fearful condition. Despite the between-group similarity on this variable, clear between-group behavioral response differences were observed in both conditions. In the Joyful condition, the children with DS shifted their attention more between the stimulus and the experimenter than did the children with WS. In addition, significantly more children with DS (75%) than children with WS (40%) produced behaviors acknowledging the joyful display and indicating that they attended to both the experimenter and the stimulus. Finally, a trend was observed for more children with DS (80%) than children with WS (50%) to approach the stimulus in the Joyful condition.

In the Fearful condition, only 25% of children with DS and 10% of children with WS demonstrated a negative response regarding the stimulus. Nonetheless, clear between-group behavioral response differences were observed. The children with DS shifted their attention between the experimenter and the stimulus more than did the children with WS. In addition, 60% of the children with DS acknowledged the experimenter’s fearful display and attended to both the experimenter and stimulus. Still, 40% of the children with DS demonstrated a positive response regarding the stimulus and significantly more children with DS than children with WS approached the stimulus without hesitation. The children with WS, on the other hand, produced very long looks at the experimenter, and significantly more children with WS (40%) than children with DS (10%) superficially imitated the experimenter’s fearful display. Only 35% of children with WS acknowledged the experimenter’s fearful display/attended to both the experimenter and the stimulus and only 25% demonstrated any response regarding the stimulus, with the majority of these children evidencing positive responses.

Taken together, these data suggest that few children in either group used the other person’s fearful response to regulate their own behavior toward an ambiguous stimulus. However, there were clear differences in the patterns of behavioral responses, as a function of diagnostic group. The results of Studies 2 to 4 help to clarify the pattern of findings from Study 1.

#### Initiating eye contact and gaze following

Children with DS performed significantly better than did children with WS in terms of both initiating eye contact and following another person’s gaze, two of the three abilities theorized to be central to the social referencing process. Both the ability to coordinate attention between a social partner and objects/events of mutual interest (initiating joint attention) and the desire to follow into the focus of another person’s attention (responding to joint attention) have been argued to facilitate social learning [70,71]. In particular, we would argue that these individual skills support the child’s emergent ability to utilize other people as a source of information about how to navigate the social and physical world. Future research focused on examining whether or not differences in joint attention abilities between children with DS and children with WS translate into group differences in social learning will provide insight into the development of socio-cognitive development in both atypical and typical development. It is also important for future studies to identify other factors that relate to social learning and how these differ between populations (typical and atypical).

Joint attention abilities also have been argued to lay the foundation for the later development of theory of mind [72,73]. Charman and colleagues [73] postulate that one possible mechanism for the relation between joint attention and theory of mind is that the child acquires theory of mind because of the experiences gained through initiations of coordinated attention between people and objects. Although there are no studies that directly compare the theory of mind abilities of children with DS and children with WS, significant delays in the development of theory of mind have been documented in both diagnostic groups [74-76]. More research is warranted to determine how the early emergence of the ability to initiate joint attention relates to the later development of theory of mind in children with these syndromes. Given the findings of early differences in joint attention ability between children with DS and children with WS, results from this line of research would contribute to the literature on the emergence of socio-cognitive development in both typical and atypical development. In addition, it is important that future studies further consider the potential long-term effects of early differences in abilities fundamental to the social referencing process on higher order social-cognitive abilities such as perspective talking and successful negotiation of conversations.

Finally, it is important to note that our findings of between-syndrome differences in joint attention favoring the children with DS were obtained even though the children with WS had significantly higher overall intellectual ability and verbal abilities than did the children with DS. These results are not the first to indicate better performance by children with DS on joint-attention related tasks when compared to CA-matched children with WS [26] despite differences in verbal and intellectual abilities favoring the children with WS [27]. Given prior findings of positive associations between TD children’s joint attention abilities and language acquisition [77-79], this may seem surprising. However, it is important to keep two things in mind. First, caregiver linguistic input provided within supported joint attention episodes is also significantly positively associated with child vocabulary acquisition [60], offering an important avenue for vocabulary development for both children with WS and DS, as well as for TD children [80]. Second, between-syndrome differences in overall intellectual ability [9,27] and in verbal short-term memory [81-83], two other factors associated with language acquisition [84,85], consistently favor children with WS [9,27,85-87]. Future studies examining all of these factors in relation to language acquisition by both children with DS and children with WS are needed to elucidate the similarities and differences in their development of language.

#### Emotional responsivity

On the task assessing the utilization of emotional reactions (Study 4), the majority of children in both groups demonstrated a response to the stimulus that was consistent with the experimenter’s response in the Joyful condition; this was not the case in the Fearful condition. While some caution should be taken interpreting the results of Study 4 due to the limited sample size, these data, once again, show that few children in either group used the experimenter’s fearful response as a source of information to regulate their own behavior toward an ambiguous stimulus. Given the prior literature documenting the difficulties of older children and adults with DS or WS in interpreting the communicative significance of facial expressions of fear (see the introduction to Study 4), the most likely explanation for the failure of most participants in both groups to use the experimenter’s fearful expression to regulate their behavior is that they did not comprehend the communicative significance of that expression. Alternatively, it is possible that because the children were engaged with a friendly and familiar experimenter, their default assumption was that the situation was not dangerous. If so, they may have reacted with curiosity about the ambiguous stimulus, regardless of the experimenter’s display. However, our finding that across Study 1 and Study 4 only a few children in each diagnostic group communicated any response to the experimenter regarding the stimulus after seeing her fearful display, indicates that at a minimum, the child’s lack of response results in a disruption in the reciprocal communicative process.

Emotional reactions are more than an index of underlying states; they are an integral component of the communicative process used as a tool to guide/influence other people’s behavior [61]. In addition, it is important to remember that social interactions are reciprocal in nature. That is, within a social interaction both people involved are simultaneously trying to communicate with and influence each other. Within these interactions, particularly adult-child interactions, emotions are frequently used to communicate social expectation regarding how to behave as well as to communicate one’s perspective on a situation to another person. As a result, when a person utilizes affective expressions to communicate information to a child, it is expected that the child will recognize this communicative act and respond accordingly. As such, even if the children in the present project disagreed with the adult’s perspective on the situation, there is a pragmatic expectation that the child respond, either verbally or nonverbally, to the adult’s communication; failure to do so presents a disruption to the social interaction. As described in the Results section of Study 1, a few children did indicate their disagreement with the experimenter’s fearful display. However, most participants either did not respond or demonstrated a positive response to the stimulus following the experimenter’s fearful expression, indicating either a failure to comprehend the significance of her expression or a pragmatic failure to communicate disagreement with the adult’s response. The potential long-term impact on emotion processing of difficulty using another person as a source of information about the surrounding world should be explored in future studies.

### Social referencing in down syndrome

When framed within the context of the social referencing process, it is plausible that, by initiating looks to the experimenter and following the experimenter’s gaze more often than do children with WS, children with DS are more likely to access the adult’s message and map it to objects and events in the environment than are children with WS. When these abilities are paired with the ability to recognize the communicative significance of a joyful expression, children with DS are able to utilize the adult as a source of information indicating that the ambiguous stimulus is enjoyable and act accordingly.

At the same time, the current findings in conjunction with the existing literature on emotion recognition in children with DS [13,64-66] indicate that, as a group, children with DS have difficulty recognizing the communicative significance of another person’s fearful reactions. This position offers a plausible explanation as to why many children with DS approached the stimulus in the Fearful condition despite frequently looking between the experimenter and the stimulus. That is, although children with DS are more likely to access the experimenter’s fearful message and map it to the stimulus than are children with WS, the children with DS still approach the fearful stimulus because they do not understand the communicative significance of the experimenter’s fearful expression. Alternatively, children with DS may have recognized the communicative significance of the experimenter’s fearful expression but assumed that the situation was not dangerous given the play context with a friendly and familiar adult. Even if this alternative explanation is correct, our finding that the majority of children with DS acted without sensitivity to the adult’s communication indicates that there are disruptions in the reciprocal communicative process. Either way, the findings from the present study highlight the importance of continued investigation of the development of social referencing by children with DS.

### Social referencing in Williams syndrome

An examination of the performance of children with WS, framed within the context of the social referencing process, indicates that it is likely that their decreased frequency of looking at adults in triadic situations and in following the adult’s gaze results in less access to information about the situation at hand and fewer opportunities to identify the source of the adult’s reaction. These disruptions cause increased confusion for the child regarding why the adult is reacting in that manner or even create situations in which the intent of the adult’s communication is completely lost. These outcomes could explain why so many children with WS did not demonstrate a response regarding the stimulus in the Fear condition in Study 1.

As pointed out by Meltzoff [88], human beings would be difficult to predict and even harder to explain if our understanding of them was restricted to their physical behaviors and movements. It is therefore possible that not identifying the source of the experimenter's reaction could be a contributing reason as to why, in Study 1, children with WS were more likely to produce very long looks to the experimenter and to superficially imitate her emotional reaction than were children with DS. This explanation could also account for why no imitation was observed in Study 4, when the source of the experimenter's reaction was much more obvious. Alternately, the lack of imitation in Study 4 could be due to children being more interested in determining what was in the box than they were in what the experimenter was doing.

Finally, the findings from Study 4, in conjunction with the existing literature [13,65,67,69], suggest that children with WS have difficulty comprehending the communicative significance of another person’s fearful expressions. As suggested above, the difficulties of children with WS in recognizing the communicative significance of emotional expressions may be impacted by their difficulties with joint attention; and even if they did understand the communicative significance of fearful expressions, our finding that most children with WS acted without sensitivity to the adult’s communication suggests that there are clear disruptions in the reciprocal communicative process for children with WS. If these hypotheses are correct, it is easy to see how breakdowns in interactions for the children with WS can occur.

It is important to note that not only does the lack of shifting attention and the reduced rate of gaze-following in triadic situations demonstrated by children with WS limit their understanding of the social interaction, but it may also influence their communicative partner’s understanding. For example, both the research assistants who coded the videotapes for this project and the experimenters who interacted with the children frequently reported that it was easier to interpret the behavioral responses of the children with DS than those of the children with WS, as the children with DS gave the adult "more information." Interestingly, this impression was obtained even though the children with WS had considerably more advanced language skills than did the children with DS, many of whom produced only a few single words or manual signs. In contrast, the coders and experimenters often said that the interactions with the children with WS were "weird" and that they were not entirely sure what was happening except that clearly something had gone wrong. Given these findings, it is important to continue the investigation of the development of social referencing in children with WS and to consider the impact of the social referencing process on the development of the phenotype.

### Impact on behavioral phenotypes

The findings obtained regarding the social referencing process and the patterns of strengths and weaknesses across the associated component abilities provide new insight into the development of the social cognitive phenotypes for these two groups of children. For children with DS, while the ability to shift attention between people and objects and the ability to follow another person’s gaze are delayed relative to TD children, these abilities are well established at a younger age than for children with WS. These abilities likely are important contributors to the social strengths demonstrated by children and adolescents with DS, and in particular to the success that children with DS have in establishing and maintaining friendships and the success that adults with DS have in securing and maintaining employment. Despite the social strengths that children with DS demonstrate, it is likely that at least young children have difficulty utilizing fearful expressions as a source of information about their environments. This pattern of being able to recognize an object as a topic of focus but not being able to utilize a communicative partner’s fearful expression could result in children inadvertently putting themselves in dangerous situations.

For children with WS, difficulties shifting attention and following the attention of others paired with a difficulty recognizing the significance of fearful expressions may help explain why very young children with WS spend so much time attending to faces [28]. Impairments in recognizing that facial expressions communicate information “about something” likely make it difficult for children with WS to make sense of human behavior in terms of underlying mental states. Basic skills such as initiating eye contact and gaze following are pivotal foundations for the development of higher order skills such as perspective taking, which are necessary to navigate the social world successfully. Accordingly, these early impairments likely alter the experiences children with WS have within their social environment, leading to significant pragmatic impairments [24], which likely lead to later difficulty establishing and maintaining friendships [20,21] and difficulty with employment [89,90] despite relative strengths in concrete vocabulary and structural language and a desire to be around other people.

It will be important to examine the development of the regulatory function of social referencing longitudinally. In addition, future studies considering the influence of other characteristics that likely differ between the DS and WS behavioral phenotypes, such as executive functioning abilities (both behavior regulation and metacognition) and temperament, on the social referencing process would be valuable.

### Neural underpinnings of social referencing

To date, no studies have systematically evaluated the neural correlates of social referencing. There are, however, some data from the literature on TD individuals elucidating the neural substrates of the abilities central to the social referencing process. Joint attention has been associated with activity in the ventromedial frontal cortex, the superior frontal gyrus, the cingulate cortex, and the caudate nuclei [91]. The anterior insula, rostral anterior cingulate cortex and the amygdala, have been associated with observing/understanding other people’s emotions [92,93].

Few studies have focused on the structure or function of any of these brain areas in individuals with DS or WS. Research on individuals with DS in comparison to CA-matched TD controls has indicated, relative to total brain gray-matter volume, no significant differences in bilateral amygdala gray-matter volume [94,95] but significantly increased gray-matter volume for bilateral caudate nucleus, bilateral frontal superior gyrus and bilateral insula [96]. Findings from studies of individuals with WS have indicated that, relative to total brain gray-matter volume, the gray matter volume of the amygdalae was significantly larger bilaterally for individuals with WS who had ID than for CA-matched TD controls [97-99], but did not differ significantly from TD CA-matched controls for individuals with WS who had low average or average IQ [100]. Structural findings for the insula have been inconsistent, with some studies of individuals with WS who had ID indicating bilateral increase [98,101] and others indicating bilateral reduction [102] relative to total brain gray-matter volume. More complex findings were reported for individuals with WS who had low average to average IQs, with bilateral reduction in dorsal anterior insula but increase in the right ventral anterior insula [103]. For individuals with WS who had low average or average IQ, the white-matter connectivity of the amygdala to the orbitofrontal cortex via the insula was compromised [103], as was the functional connectivity of the amygdala to the orbitofrontal cortex during emotional processing tasks [100]. Alteration in amygdala functioning and regulation was also found for individuals with WS who had ID [104]. Research documenting similarities and differences in the neuropathology associated with DS and with WS that is focused on brain regions and pathways hypothesized to play a role in social referencing is needed to provide further information regarding the neuropathology underlying social referencing for these two syndromes.

## Conclusions

The present project was the first to examine the regulatory function of social referencing in two genetically well-defined neurodevelopmental disorders (DS and WS) characterized by differing socio-cognitive phenotypes and to consider the influence of phenotypic group differences on the abilities fundamental to social referencing and on the social referencing process itself. A syndrome-specific approach to the examination of the social referencing process offers the opportunity to consider the influences of specific abilities fundamental to the development of social cognition. The findings from the present project demonstrate that the behavioral responses of children with DS and children with WS differ during the social referencing process. These differences are likely, in part, a result of differences in initiating eye contact in triadic situations and in following another person’s gaze. Both children with DS and children with WS evidenced difficulty utilizing the communicative significance of fearful expressions. These early differences between children with DS and children with WS likely alter the social experiences of these two groups of children thereby contributing to the clear differences observed in these behavioral phenotypes and in adult outcomes. Future studies focused on characterizing factors contributing to these early socio-cognitive differences and on their long-term impact will allow for further specification of the mechanisms associated with socio-cognitive abilities, which in turn may be used to refine developmental models of social cognition in both typical and atypical development.

## Abbreviations

AE: Age-equivalent; CA: Chronological age; DAS-II-EY: Differential Ability Scales II Early Years; DD: Developmental delay; DQ: Developmental quotient; DS: Down syndrome; ELA: Expressive language age; ID: Intellectual disability; MA: Mental age; PPVT-4: Peabody Picture Vocabulary Test 4th edition; RLA: Receptive language age; SSs: Standard scores; TD: Typically developing; WS: Williams syndrome.

## Competing interests

Both authors state that there are no actual or potential conflicts of interest, including financial or non-financial relationships within five years of beginning this work that could inappropriately influence, or be perceived to influence, their work.

## Authors’ contributions

AJT conceptualized the study and had primary responsibility for task design, data analysis and interpretation, and drafting of the manuscript. CBM participated in the design of the study, provided data interpretation and support, and edited the manuscript. Both authors read and approved the final manuscript.
